# Medical Legislation in Texas

**Published:** 1895-01

**Authors:** L. S. Downs

**Affiliations:** Galveston


### Medical Legislation in Texas.

GALVESTON, December 10, 1894.

*Editor Texas Medical Journal:*

I have read Dr. Wooten's article, and your excellent editorial comments on medical legislation in Texas.

If you will allow me space in your excellent JOURNAL I would be pleased to say a few words on the subject from an Eclectic point of view. We are, and have always been, heartily in favor of proper medical laws. We deem it but right and just, not only to the profession, but to the laity, to have a more rigid supervision by the State, over the practice of medicine. It is our desire that the requirement be raised to the highest limit possible.

While I was President of our State Association I addressed Dr. Sears a letter upon the subject of medical legislation, and urged the propriety of taking some concerted action in the matter, by the presidents of the three schools. The doctor was cordial in his reply, and said he would be pleased to lend his influence to so laudable an undertaking. And later he thought best to defer action till after the annual meeting of his Association at Austin. No further correspondence was had on the matter.

Feeling that some progressive movement was demanded from the exigencies of the case, at the last meeting of our State Association, a legislative committee was appointed to prepare a bill to be placed before our next legislature, with the proper influences accompanying, to cause an early and unbiased consideration of the same, by that body.

Our committee will be pleased to confer with like committee from the other schools of the State, and formulate a law that will be equitable and fair to all parties concerned, a credit to the State, and a benefit to the profession.

Your fraternally,

L. S. DOWNS, M. D.

